# Impact of an Online Risk Calculator for Sentinel Node Positivity on Management of Patients with T1 and T2 Melanomas

**DOI:** 10.1245/s10434-024-15456-w

**Published:** 2024-05-27

**Authors:** Alec A. Winder, Zoe Boyer, Sydney Ch’ng, Jonathan R. Stretch, Robyn P. M. Saw, Kerwin F. Shannon, Thomas E. Pennington, Omgo E. Nieweg, Alexander H. R. Varey, Richard A. Scolyer, John F. Thompson, Anne E. Cust, Serigne N. Lo, Andrew J. Spillane, Andrea L. Smith

**Affiliations:** 1 Melanoma Institute Australia, The University of Sydney, Sydney, NSW Australia; 2 Faculty of Medicine, Health and Human Sciences, Macquarie University, Sydney, NSW Australia; 3 Faculty Medicine and Health, The University of Sydney, Sydney, NSW Australia; 4 The Daffodil Centre, University of Sydney, A Joint Venture with Cancer Council NSW, Sydney, NSW Australia; 5 Tissue Pathology and Diagnostic Oncology, Royal Prince Alfred Hospital and NSW Health Pathology, Sydney, NSW Australia; 6 Charles Perkins Centre, The University of Sydney, Sydney, NSW Australia; 7 Royal Prince Alfred Hospital, Sydney, NSW Australia; 8 Royal North Shore Hospital, Sydney, NSW Australia; 9 Mater Hospital, Sydney, NSW Australia

## Abstract

**Background:**

Predicting which patients with American Joint Committee on Cancer (AJCC) T1–T2 melanomas will have a positive sentinel lymph node (SLN) is challenging. Melanoma Institute Australia (MIA) developed an internationally validated SLN metastatic risk calculator. This study evaluated the nomogram’s impact on T1–T2 melanoma patient management at MIA.

**Methods:**

SLN biopsy (SLNB) rates were compared for the pre- and post-nomogram periods of 1 July 2018–30 June 2019 and 1 August 2020–31 July 2021, respectively.

**Results:**

Overall, 850 patients were identified (pre-nomogram, 383; post-nomogram, 467). SLNB was performed in 29.0% of patients in the pre-nomogram group and 34.5% in the post-nomogram group (*p* = 0.091). The overall positivity rate was 16.2% in the pre-nomogram group and 14.9% in the post-nomogram group (*p* = 0.223). SLNB was performed less frequently in T1a melanoma patients in the pre-nomogram group (1.1%, *n* = 2/177) than in the post-nomogram group (8.6%, *n* = 17/198) [*p* ≤ 0.001]. This increase was particularly for melanomas with a risk score ≥ 5%, with an SLN positivity rate of 11.8% in the post-nomogram group (*p* = 0.004) compared with zero. For T1b melanomas with a risk score of > 10%, the SLNB rate was 40.0% (8/20) pre-nomogram and 75.0% (12/16) post-nomogram (*p* = 0.049).

**Conclusions:**

In this specialized center, the SLN risk calculator appears to influence practice for melanomas previously considered low risk for metastasis, with increased use of SLNB for T1a and higher-risk T1b melanomas. Further evaluation is required across broader practice settings. Melanoma management guidelines could be updated to incorporate the availability of nomograms to better select patients for SLNB than previous criteria.

**Supplementary Information:**

The online version contains supplementary material available at 10.1245/s10434-024-15456-w.

Sentinel lymph node biopsy (SLNB) identifies occult metastatic melanoma, improves regional disease control, and provides important staging and prognostic information.^1^ This information can guide management decisions regarding follow-up and selection for adjuvant systemic therapy.^2^ On multivariable analysis, the Multicentre Selective Lymphadenectomy Trial-I showed that SLN positivity was the strongest predictor of melanoma-specific survival (MSS) for melanomas > 1 mm thick. For intermediate-thickness melanomas (1.2–3.5 mm) with metastatic disease within an SLN, early intervention with SLNB may be associated with increased MSS compared with nodal observation.^1^ The American Joint Committee on Cancer (AJCC) 8th Edition tumor staging guidelines define T1a disease as melanomas < 0.8 mm and non-ulcerated, while T1b melanomas are non-ulcerated tumors 0.8–1.0 mm thick or ulcerated tumors ≤ 1 mm thick,^3^ and T2a melanomas are > 1–2 mm thick without ulceration and T2b melanomas are > 1–2 mm with ulceration. Current Australian Melanoma Management Guidelines recommend that SLNB be considered for all patients with melanomas ≥ 1 mm thick and for patients with melanomas > 0.75–1.0 mm thick with other high-risk pathological features,^2^ which is similar to National Comprehensive Cancer Network (NCCN) guidelines.^4^

Predicting tumor metastasis for thin melanomas remains difficult, with a recent meta-analysis showing high rates of negative SLNBs (overall 94.4%).^5^ Although SLNB is generally a safe procedure, the overall complication rate is approximately 11%, but most complications are minor.^6^ In some elderly patients or those with comorbidities, SLNB is omitted and lymphoscintigraphy (LSG) with focused ultrasound monitoring of the SLNs is used instead. One study found that patients undergoing ultrasound monitoring had similar rates of MSS, overall recurrence-free survival (RFS), and distant RFS as those managed with SLNB, however regional RFS was worse if SLNB was not performed.^7^

To better stratify the risk of individual patients having SLN metastasis, the Memorial Sloan Kettering Cancer Center (MSKCC) published an SLN metastasis nomogram in 2005.^8^ Subsequently, Melanoma Institute Australia (MIA) developed a refined risk calculator, made publicly available in August 2020 (www.melanomarisk.org.au). Modeling demonstrated that the MIA calculator, intended as an adjunct to assist with clinical decision making, significantly improved accuracy for predicting SLN positivity (73.9%) compared with the MSKCC model (67.7%).^9^ As a guide, patients with a < 5% risk of a positive SLN should not have SLNB, those with a 5–10% risk should consider SLNB, and those with a > 10% risk are encouraged to have SLNB. The NCCN guidelines make similar recommendations for SLNB when risk scores are > 10%.^4^ It was anticipated that the MIA risk calculator could potentially reduce unnecessary SLNBs in low-risk patients with T1b/T2 tumors, and detect positive SLNs in patients with T1a melanomas in whom SLNB is usually not performed. If this was the only mechanism for selecting patients for SLNB and other factors were ignored, this would be expected to increase the positivity rate in the T1b/T2 group, although this may be canceled out by increased procedures on patients from the T1a group.

Our primary aim was to determine whether the introduction of the MIA risk calculator was associated with a change in clinical practice at MIA by reporting rates of SLNB in patients with T1–T2 melanomas before and after the calculator became available. The secondary aims were to determine whether, over the same period, there had been an impact on the rates of SLN positivity and the utilization of focused ultrasound monitoring rather than SLNB after LSG for patients with T1–T2 melanomas.

## Methods

### Data Collection

Two patient groups were identified—a pre-nomogram group and a post-nomogram group. The MIA risk calculator was made available to the public in August 2020 but was available to MIA clinicians for several months prior to this. To avoid cross-contamination, patients were included from 1 July 2018 to 30 June 2019 for the pre-nomogram group and from 1 August 2020–31 July 2021 for the post-nomogram group. Surgeons at MIA were surveyed on their experience using the nomogram (Online Resource 1).

Data for the pre-nomogram group were obtained from the MIA research database, and for the post-nomogram group, from individual surgeons’ data as these data had not yet been incorporated into the database. Data extracted for the pre- and post-nomogram groups included age at melanoma diagnosis, sex, comorbidities, site of melanoma, Breslow thickness, melanoma subtype, Clark level, mitotic rate, ulceration, and lymphovascular invasion. Subsequent treatment information obtained included SLNB, number of nodes removed, number of positive SLNs, any recurrence data, and complications of the procedure. All primary melanoma pathology reports were reviewed by MIA pathologists, with no changes in pathological protocols being made over the study period. Immunohistochemistry was routinely used in SLN assessment. Lymph nodes were defined as positive if evidence of metastatic melanoma was detected.

### Inclusion Criteria

Patients were included in the study if they had a melanoma 0.1–2 mm thick and one of the five melanoma subtypes listed on the MIA risk calculator (superficial spreading melanoma, nodular melanoma, lentigo maligna melanoma, acral melanoma, and pure desmoplastic [> 90%] melanoma).^9^ Patients with multiple T1-2 melanomas or a prior history of melanoma were also included. Patients whose melanoma subtype was not documented or who had a subtype other than those listed above were excluded; in total, 77 patients in the pre-nomogram group and 90 patients in the post-nomogram group were excluded.

### Comorbidity

The Charlson comorbidity index,^10^ a predictor of 10-year survival, was determined for each patient using mdcalc.com to better compare the comorbidities of both groups.

### Risk of Sentinel Lymph Node (SLN) Metastasis

The SLN metastasis risk score was calculated for each patient using the MIA risk prediction tool found at melanomarisk.org.au. Risk scores were divided into very low risk (< 5% of lymph node metastasis), low risk (5–10%), and higher risk > 10%).

### Statistical Analysis

Statistical analysis was performed using SPSS version 24 (IBM Corporation, Armonk, NY, USA). Between-group differences in categorical variables were compared using the Chi-square test; where small numbers of patients were involved, Fisher’s exact test was used. Continuous variables were summarized using median (range), stratified by group. Mean differences between groups were tested using a t-test, and median differences were tested using the Mann–Whitney U test. Logistic regression analysis was performed to evaluate factors associated with the performance of SLNB between groups (pre- and post-nomogram) within each T-stage category. The significance value was determined to be < 0.05 for all calculations using a two-sided test.

### Ethics

Ethics approval was granted by the MIA Research Committee (study number MIA2020/314) and the Sydney Local Health District Ethics Committee (#X15-0311).

## Results

### Demographics

In total, 383 patients were included in the pre-nomogram group (164 females, 219 males) and 467 in the post-nomogram group (201 females, 266 males). The median age in the pre- and post-nomogram groups was 62 years (13–94 years) and 62 years (14–93 years), respectively (*p* = 0.844). There were no significant differences between the groups (Table 1), except a greater proportion of patients in the pre-nomogram group had a past history of melanoma compared with the post-nomogram group (*n* = 63/383, 16.4% vs. *n* = 39/467, 8.4%) (*p* = 0.001).

**Table 1:** Demographics for pre- and post-nomogram patients

	Pre-nomogram	Post-nomogram	*p*-Value^a^
Number of patients	383	467	–
Age, years [median (range)]	62 (13–94)	62 (14–93)	0.844
Female:male ratio	43:57	43:57	0.950
CCM index [median (range)]	2 (0–11)	2 (0–7)	0.481
Previous melanoma [*n* (%)]	63 (16.4)	39 (8.4)	0.001^b^

*CCM* Charlson comorbidity

^a^Based on t-test for means difference, Mann–Whitney U test for median difference, and Chi-square for categorical variables

^b^*p* < 0.05 indicates significance

### Tumor Characteristics

The median Breslow thickness was 0.9 mm, median Clark level was 3, and median mitotic rate was 1 per mm^2^ in both groups (Table 2). In the pre-nomogram group, 22 tumors (5.7%) were ulcerated compared with 43 (9.2%) in the post-nomogram group (*p* = 0.088). There were no significant differences in melanoma subtypes (*p* = 0.551) or melanoma sites (*p* = 0.100) between the two groups.

**Table 2:** Tumor characteristics for pre- and post-nomogram patients

	Pre-nomogram	Post-nomogram	*p*-Value^a^
Number of patients	383	467	
Lymph node biopsy (%)	111 (30.0)	161 (34.5)	0.091
Breslow thickness, mm (median)	0.9	0.9	0.144
T1a	177	199	
T1b	78	85	
T2a	110	164	
T2b	18	19	
Clark level (median)	3	3	0.970
Ulceration present (%)	22 (5.7)	43 (9.2)	0.088
Lymphovascular invasion present (%)	5 (1.4)	4 (0.9)	0.482
Mitotic rate per mm^2^ [median] (%)	1	1	0.781
0	163 (45.9)	230 (49.3)	
1 per mm^2^	78 (22.0)	78 (16.7)	
2 per mm^2^	36 (10.1)	50 (10.7)	
3 per mm^2^	23 (6.5)	38 (8.1)	
4+	55 ((15.5)	64 (13.7)	
Melanoma subtype (%)			0.551
Superficial spreading	293 (76.5)	351 (75.2)	
Nodular	37 (9.7)	54 (11.6)	
Lentigo maligna	48 (12.5)	49 (10.5)	
Acral	2 (0.5)	4 (0.9)	
Desmoplastic	3 (0.8)	9 (1.9)	
Involved margins on biopsy (%)	163 (42.6)	116 (40.3)	0.747
Site (%)			0.100
Head/neck	73 (19.3)	95 (20.3)	
Trunk	160 (41.8)	151 (32.3)	
Upper limb	74 (19.3)	105 (22.5)	
Lower limb	76 (19.8)	116 (24.8)	

^a^Based on t-test for means difference, Mann–Whitney U test for median difference, and Chi-square for categorical variables

### Sentinel Lymph Node Biopsy (SLNB) Rates

Overall, there was no significant difference in the rate of SLNB between the two groups (*p* = 0.088) [Table 3]. In the pre- and post-nomogram groups, 111 (29.0%) and 161 (34.5%) patients, respectively, underwent SLNB. There was no significant difference in the rate of SLNB positivity, median age, or Charlson comorbidity index between the two groups (Table 3). The rates of SLNB for patients < 40 years of age were 28.9% (*n* = 13/45) in the pre-nomogram group and 46.2% (*n* = 24/52) in the post-nomogram group, but the difference was not statistically significant (*p* = 0.080).

**Table 3:** Sentinel lymph node biopsy rates for pre- and post-nomogram patients

	Pre-nomogram	Post-nomogram	*p*-value^a^
Total patients	383	467	
SLNB performed (%)	111 (29.0)	161 (34.5)	0.088
Positive SLNB (%)	18 (16.2)	24 (14.9)	0.773
Age, years [median]	57.8 (13–83)	55.6 (15–88)	0.340
Age ≤ 40 years (%)	13/45 (28.9)	24/52 (46.2)	0.080
Age ≥ 80 years (%)	2/36 (5.6)	4/48 (8.3)	0.625
CCM index average (range)	1.81 (0–9)	1.61 (0–6)	0.112

*SLNB* sentinel lymph node biopsy, *CCM* Charlson co-morbidity

^a^Based on t-test for means difference, Mann–Whitney U test for median difference, and Chi-square for categorical variables

### Sentinel Node Biopsy and T Stage

There were no significant differences in the rates of SLNB for T1b, T2a, and T2b melanoma patients overall (Table 4). However, the proportion of patients undergoing SLNB was lower in the pre-nomogram group for T1a melanomas compared with the post-nomogram group (2/177 pre-nomogram vs. 17/198 post-nomogram; odds ratio [OR] 1.08, 95% confidence interval [CI] 1.03–1.13; *p* ≤ 0.001). When an interaction test between T stage and the pre- and post-nomogram groups was included in the logistic model, the effect was not statistically significant (*p* = 0.492), suggesting the impact of the MIA risk calculator was not significantly different between T-stage groups, and the increased SLNB rate for T1a melanomas could be due to chance.

**Table 4:** Rate of sentinel lymph node biopsy for T stage of melanoma

Stage	Pre-nomogram SLNB (%)	Post-nomogram SLNB (%)	OR (95% CI)	*p*-value^a^
T1a	2/177 (1.1)	17/198 (8.6)	1.08 (1.03–1.13)	< 0.001^b^
T1b	24/78 (30.8)	37/86 (43.0)	1.13 (0.98–1.31)	0.106
T2a	74/111 (66.7)	90/164 (54.9)	0.89 (0.79–1.0)	0.051
T2b	11/17 (64.7)	17/19 (89.5)	1.28 (0.98–1.67)	0.078
Total	111/383 (29.0)	161/467 (34.5)	1.05 (0.99–1.13)	0.088

*CI* confidence interval, *OR* odds ratio, *SLNB* sentinel lymph node biopsy

^a^Based on t-test for means difference, Mann–Whitney U test for median difference, and Chi-square for categorical variables

^b^*p* < 0.05 indicates significance

Each T stage was divided into risk categories (very low risk, < 5%; low risk, 5–10%; higher risk, > 10%) [Table 5]. Patients with low- and higher-risk melanomas in the T1a pre-nomogram group were less likely to undergo SLNB than those in the post-nomogram group. For low-risk T1a melanomas, 2/54 (3.7%) patients underwent SLNB pre-nomogram versus 12/76 (15.8%) post-nomogram (*p* = 0.042). For higher-risk T1a melanomas, 0/14 patients underwent SLNB pre-nomogram versus 5/14 (35.7%) post-nomogram (*p* = 0.041). For higher-risk T1b melanomas (risk score > 10%), the rate of SLNB was 40.0% (8/20) in the pre-nomogram group and 75.0% (12/16) in the post-nomogram group (*p* = 0.049).

**Table 5:** Rates for sentinel lymph node biopsy, lymphoscintigraphy alone (with ultrasound follow-up), and both investigation groups combined for all T stages and risk of LN metastasis

	Very low risk < 5%			Low risk 5–10%			Higher risk > 10%		
	Pre-nomogram	Post-nomogram	*p*-value	Pre-nomogram	Post-nomogram	*p*-value	Pre-nomogram	Post-nomogram	*p*-value
T1a									
SLNB (%)	0	0	–	2 (3.7)	12 (15.8)	0.042^a^	0	5 (35.7)	0.041^a^
LSG only (%)	5 (4.6)	6 (5.6)	0.768	2/52 (3.8)	25/64 (39.1)	< 0.001^a^	0	2/9 (22.2)	0.142
LSG + SLNB (%)	5 (4.6)	6 (5.6)	0.768	4 (7.5)	37 (48.7)	< 0.001^a^	0	7 (50.0)	0.006^a^
Total	109	108		54	76		14	14	
T1b									
SLNB (%)	3 (18.8)	7 (28.0)	0.712	13 (31.0)	18 (40.0)	0.502	8 (40.0)	12 (75.0)	0.049^a^
LSG only (%)	4/13 (30.8)	2/18 (11.1)	0.208	14/29 (48.3)	16/27 (59.3)	0.436	5/12 (41.7)	4/4 (100)	0.088
LSG + SLNB (%)	7 (43.8)	9 (36.0)	0.746	27 (64.3)	34 (75.5)	0.349	13 (65.0)	16 (100)	0.011^a^
Total	16	25		42	45		20	16	
T2a									
SLNB (%)	6 (42.8)	6 (18.2)	0.140	35 (62.5)	44 (53.0)	0.298	33 (80.5)	40 (83.3)	0.786
LSG only (%)	3/8 (37.5)	15/27 (55.6)	0.443	10/21 (47.6)	35/39 (89.7)	0.001^a^	5/8 (62.5)	7/8 (87.5)	0.569
LSG + SLNB (%)	9 (64.3)	21 (63.6)	1	45 (80.4)	79 (95.2)	0.010^a^	38 (92.3)	47 (97.9)	0.331
Total	14	33		56	83		41	48	
T2b									
SLNB (%)	0	0	–	1 (33.3)	4 (80.0)	0.464	10 (71.4)	13 (92.9)	0.326
LSG only (%)	0	0	–	1/2 (50)	1/1 (100)	1	3/4 (75)	1/1 (100)	1
LSG + SLNB (%)	0	0	–	1 (33.0)	5 (100)	0.375	13 (92.9)	14 (100)	1
Total				3	5		14	14	

*SLNB* sentinel lymph node biopsy, *LSG* lymphoscintigraphy

^a^*p* <  0.05 indicates significance

For the T2a and T2b melanomas, no significant differences were noted between the pre- and post-nomogram cohorts. There was however a non-significant reduction in the number of very-low-risk patients undergoing SLNB in the T2a post-nomogram group (6/33, 18.2%) compared with the pre-nomogram group (6/14, 42.8%) [*p* = 0.140]. None of the T2b patients were deemed to be very low risk of SLN metastasis and the overall numbers in this T2b group was low in both cohorts.

### Sentinel Node Positivity Rates

The SLN positivity rate overall was 16.2% (*n* = 18/111) in the pre-nomogram group and 14.9% (*n* = 24/161) in the post-nomogram group (*p* = 0.223) [Table 6]. For T1a melanomas, the overall rate of positivity was increased in the post-nomogram group from 0/2 pre-nomogram to 2/17 (11.8%) post-nomogram (*p* = 0.004). The overall rate of SLN positivity was not affected for T1b or T2 melanomas. Although not significant, the rate of positivity for T2b melanomas was higher pre-nomogram 3/11 (27.3%) versus 1/17 (5.9%) post-nomogram (*p* = 0.062).

**Table 6:** Sentinel node positivity rates for T stages and risk categories based on the risk calculator

	Very low risk < 5%		Low risk 5–10%		Higher risk > 10%		Total		
	Pre-nomogram	Post-nomogram	Pre-nomogram	Post-nomogram	Pre-nomogram	Post-nomogram	Pre-nomogram	Post-nomogram	*p*-value
T1a	0/0^a^	0/0	0/2	2/12 (16.7)	0/0	0/5 (0)	0/2	2/17 (11.8)	0.004^b^
T1b	0/3	0/7	1/13 (8.0)	1/18 (5.6)	1/8 (12.5)	2/12 (16.7)	2/24 (8.3)	3/37 (8.1)	0.268
T2a	0/6	1/6 (16.7)	3/35 (8.6)	5/44 (11.4)	10/33 (30.3)	12/40 (30.0)	13/74 (17.6)	18/90 (20.0)	0.136
T2b	0/0	0/0	0/1	0/4	3/10 (30.0)	1/13 (7.7)	3/11 (27.3)	1/17 (5.9)	0.062
Total	0/9	1/13 (7.7)	2/38	7/69 (10.1)	16/64 (25)	16/79 (20.1)	18/111 (16.2)	24/161 (14.9)	0.223

^a^Data are expressed as number of positive sentinel node procedures/number of sentinel node procedures (percentage)

^b^*p* < 0.05 indicates significance

### Lymphoscintigraphy and Focused Ultrasound Monitoring, Without SLNB

The use of LSG and focused ultrasound monitoring (without SLNB) increased from 52/272 (19.1%) patients in the pre-nomogram group to 114/306 (37.3%) patients in the post-nomogram group (*p* ≤ 0.001). This increase was most evident in the T1a melanoma group, [7/175 (4.0%) patients pre-nomogram and 33/181 (18.2%) patients post-nomogram; *p* ≤ 0.001] and the T2a melanoma group [18/37 (48.6%) patients pre-nomogram and 57/74 (77.0%) patients post-nomogram; *p* = 0.05] (Table 5). Subdividing the melanoma T stages by predicted risk of SLN metastasis, this increase seemed to be related mostly to low-risk melanomas (5–10% risk of SLN metastasis). For T1a nomogram-predicted low-risk melanomas, the rate of LSG without SLNB increased from 2/52 (3.8%) patients pre-nomogram to 25/64 (39.1%) patients post-nomogram (*p* ≤ 0.001), and for T2a nomogram-predicted low-risk melanomas, LSG without SLNB increased from 10/21 (47.6%) patients to 35/39 (89.7%) patients (*p* = 0.001). The other T stages and risk categories were not affected.

Patients aged ≥ 80 years or with a Charlson comorbidity score of ≥ 4 were more likely to undergo LSG with ultrasound follow-up than LSG with SLNB in both the pre- and post-nomogram groups compared with younger and healthier patients. For patients undergoing staging investigations post-nomogram who were < 80 years of age, 94/252 (37.3%) had LSG and ultrasound monitoring without SLNB, but for patients ≥ 80 years of age, 20/24 (83.3%) had LSG and ultrasound monitoring without SLNB (*p* ≤ 0.001). For patients with a Charlson comorbidity score of < 4, 86/234 (36.8%) had LSG without SLNB, but for those with a score of ≥ 4, 28/41 (68.3%) had LSG without SLNB (*p* ≤ 0.001).

### Complications

The overall complication rate for SLNB in both groups was 21/272 (7.7%) patients, including infections, seromas and lymphedema in one patient. Complications developed in 8/111 (7.2%) patients in the pre-nomogram group over an average 16.3 months and 13/161 (8.1%) patients (*p* = 0.792) in the post-nomogram group. Data in the post-nomogram group were collected prospectively from individual surgeons’ databases and represent the acute complications of surgery only.

### Nodal Recurrence

For the pre-nomogram group, follow-up was 27.2 months (21.8–34.4 months). Seven patients developed nodal recurrence—2/93 after negative sentinel node biopsies, 2/52 in patients who had lymphatic mapping without SNB detected with ultrasound monitoring, and 3/221 in patients who were monitored clinically. Two patients had T1b melanomas, three had T2a melanomas, and two had T2b melanomas. For the post-nomogram group, data were collected prospectively from individual surgeons’ databases, with insufficient follow-up to be meaningful.

## Discussion

The overall rates of SLNB and SLN positivity were not significantly different after the introduction of the nomogram. In T1a melanoma patients, the overall rate of SLNB was significantly increased in the post-nomogram group but with a higher rate of nodal positivity (0% vs. 11.8%), which is higher than in several other published studies.^5,11^ This may suggest that the nomogram is helping to identify a higher risk subset of these thin melanomas; however, with very low numbers (2), this could still be by chance. This result is consistent with the survey that found most of the surgeons used the MIA-nomogram frequently, and it affected their recommendations, particularly for thin melanomas and younger patients, who are at greater risk of nodal metastasis.^11^ For T1b melanomas, the overall rate of SLNB was non-significantly higher in the post-nomogram group (30.8% vs. 43%); however, for higher-risk T1b melanomas (risk score > 10%), the rate of SLNB was 40.0% (8/20) in the pre-nomogram group and 75.0% (12/16) in the post-nomogram group (*p* = 0.049).

For the T2a and T2b melanomas, no significant differences were noted between the pre- and post-nomogram cohorts. However, there was a non-significant reduction in the number of very-low-risk patients undergoing SLNB in the T2a post-nomogram group, i.e. 6/14 (42.8%) versus 6/33 (18.2%) [*p* = 0.140]. None of the T2b patients were deemed to be at very low risk of SLN metastasis, and the overall numbers in this group were low. However, all these results must be interpreted with caution due to low numbers and that the interactions test by T stage was non-significant, thus further data are needed to establish the impact on clinical practice.

The aim of the MIA risk calculator is to more accurately predict an individual patient’s risk of nodal metastasis and facilitate discussions about the pros and cons of SLNB by giving more concrete guidance on the likelihood of a positive SN for that individual. It was hoped that this would potentially translate to a reduction in unnecessary surgery and an increase in the SLN positivity rate. However, over this initial period of 1 year, there was a non-significant increase in the rate of SLNB performed (*p* = 0.088) and a non-significant decrease in SLNB positivity (*p* = 0.112). This in part seems to be related to the surprisingly low rate of nodal positivity in the T2b post-nomogram group, likely a statistical aberration related to low numbers of patients. Second, aiming to avoid missing patients previously seen as very low risk of nodal metastasis according to Australian guidelines criteria has resulted in 8.6% of T1a patients undergoing SLNB, where previously, SLNB was rarely performed (1.1%). The MIA nomogram guidelines (and NCCN guidelines) recommend discussing SLNB with patients who have a risk score of 5–10%, and this may push many patients into wanting surgery when the nomogram demonstrates this outcome. If either the current Australian guidelines or the nomogram suggest SLNB to be appropriate, the patient may be offered surgery and thus increase the number of procedures being performed overall. Statistically, the MIA risk calculator should improve the nodal positivity rate compared with current guidelines, however this is in isolation, and, in reality, decision making is a more complex process.

Other factors that may have contributed to the increase in patients with T1a melanomas undergoing SLNB include the initial MIA risk calculator version’s wider CIs, as only 10.8% of the patients used in the development cohort had melanomas < 1 mm (376/3477).^9^ Lower patient numbers led to fewer data points and wider CIs for patients with thin melanomas in the MIA SLN predictive model compared with patients with thicker melanomas. Of interest, the MD Anderson dataset, used to validate the MIA nomogram, had 1357 T1 melanomas. When these data were used to compare the MIA and MSKCC nomograms, the MIA nomogram performed better at predicting SLN positivity.^9^ A recent nomogram published by Maurichi et al.^12^ looking at the risk of SLN metastasis for T1 melanomas incorporated 3666 patients, almost 10 times as many as used in the MIA risk calculator for the same thickness of melanoma, and therefore CIs will likely be smaller in their data. The MIA nomogram has subsequently been further validated, resulting in narrowed CI, but, reassuringly, the point estimates remained very similar (unpublished data).

In the future, better biomarkers or prognostic gene expression profiles (GEPs) may provide enough information for prognostication and treatment planning to obviate the need for SLNB. GEP testing was not performed for any of the patients in this study, and at present its value has not been established in prospective clinical trials without SLNB. The tests remain costly and may be up to around US$7000. GEP testing may provide additional information over standard clinicopathological features by stratifying patients into high- and low-risk groups. Use of GEP tests has been found to alter recommendations for patient follow-up, imaging, and adjuvant trials.^13^ GEP testing may be particularly beneficial for patients with thin- to intermediate-thickness melanomas, with a negative sentinel node biopsy. Currently, the MIA nomogram is freely available and provides more accurate prediction of SLN metastasis than other nomograms and guideline recommendations alone.^9^

There was an increase in the use of LSG and ultrasound monitoring without performing SLNB in the post-nomogram group, particularly for patients with low-risk melanomas (5–10% risk) who had T1a and T2a disease. It is noteworthy that some individual surgeons used this strategy more often whereas others rarely did. The decision is likely not solely related to availability of the risk calculator, as it was used pre-nomogram. Ipenburg et al.^7^ reported use of this strategy for elderly patients and those with comorbidities, as the risks of complications from surgery are higher and the likelihood of nodal metastasis are less in the elderly. Observed patients who underwent therapeutic node dissection were noted to have significantly more involved nodes compared with SN-positive patients who underwent completion lymph node dissection. Prior research has suggested that survival correlates inversely with the number of involved nodes. This may represent a potential risk of this strategy, although survival was not affected in the study, possibly due to sample size. In the present study, LSG with ultrasound monitoring only was used more frequently for elderly patients and those with comorbidities. Other reasons for its use included patients who had a low risk of SN positivity on the nomogram but met the Australian guidelines threshold for recommending SLNB, or the identification of complex lymphatic mapping with drainage to multiple node fields, making the risk/benefit balance in favor of not proceeding with the SLNB. As ultrasound monitoring involves active monitoring and not potentially morbid nodal surgery for low-risk melanomas, this may be seen as an attractive option by both the clinician and the patient. However, ultrasound monitoring adds additional costs over time and potential unwarranted anxiety compared with observation alone.

### Limitations

As this is an initial review of the use of the nomogram at MIA, the number of patients overall is reasonably large, but with subgroup analysis and low SN-positivity rates in early melanoma, numbers become relatively small. This means that minor but significant alterations in practice may go unnoticed and the risk of sampling error is increased. This is a before and after study, not a randomized study, therefore other factors not evident in the data could have affected the results: changes in practice occur over time, as do clinicians’ preferences. Other factors affect clinical decisions, such as national guidelines, patients’ characteristics, i.e. age, comorbidities, previous history of melanoma or SLNB, as well as patient preferences. Whether and how such factors were considered when surgeons were considering recommending SLNB could not be evaluated on a systematic basis in this study. Another major limitation of this study is a lack of long-term outcomes for these patients. Data on false-negative SLNB and long-term regional recurrence rates in patients who did not undergo SLNB could help to determine if appropriate patients are being selected for SLNB. Recurrence data were not collected for this study as the aim was not to validate the risk tool, however this information may also influence clinical decision making.

Lastly, these data were collected from a highly specialized melanoma treatment center, therefore effects on local practice may not reflect changes in practice generally.

## Conclusions

SLNB remains an important tool for staging and determining prognostic information for patients with primary melanomas. This study of an individual institutions’ experience shows the introduction of the MIA nomogram was associated with an increased rate of SLNB for T1a melanomas, with indications of an increase in SLN metastasis detection for this group previously seen as very low risk. The decision to perform SLNB is complex and likely impacted by the nomogram, current guidelines, and individual patient fitness and preferences.

The overall rates of SLNB and SLN-positivity were unchanged by the introduction of the MIA nomogram in this initial study period, but increased use of LSG and ultrasound monitoring was seen. Larger patient numbers will be needed to confirm the nomogram’s true effect on clinical practice.

### Supplementary Information

Below is the link to the electronic supplementary material.

Supplementary file1 (DOCX 14 kb)
