# Supplementary material for: Impact of an Online Risk Calculator for Sentinel Node Positivity on Management of Patients with T1 and T2 Melanomas
Source: Ann Surg Oncol. 2024 May 27;31(8):5331–9. doi: 10.1245/s10434-024-15456-w (PMC11236927; doi:10.1245/s10434-024-15456-w)
Supplement: Supplementary file 1 — Supplementary file1 (DOCX 14 kb) [file 10434_2024_15456_MOESM1_ESM.docx]

## **ONLINE RESOURCE 1**

Eight surgeons surveyed

- Q1 Did you use the MIA nomogram (routinely, frequently, occasionally, rarely or not at all)?
  - Response 1: Two routinely, six frequently.
- Q2 If so, did the nomogram influence your recommendations for SLNB (not at all, slightly, moderately, greatly or totally)?
  - Response 2: Four surgeons reported greatly: one often; two slightly; and one almost never for T1b or thicker, occasionally for T1a but not thinner than 0.5 mm. Five surgeons indicated that the calculator most frequently influenced the recommendation of whether or not to proceed to a SLNB for thinner melanomas, younger patients and the elderly.
- Q3 Did you use any nomogram prior to the MIA risk calculator (routinely, frequently, occasionally, rarely or not at all)?
  - Response 3: One surgeon reported frequently; two occasionally; two rarely; and three never.
- Q4 If so, did that non-MIA nomogram influence your recommendations for SLNB (not at all, slightly, moderately, greatly or totally)?
  - Response 4: Greatly in one surgeon; one moderately in one; rarely in one; and for two surgeons it never influenced their recommendation.
